# Case of Strangulation of a Portion of the Intestines from Rupture of the Mesentery

**Published:** 1819-02

**Authors:** Richard Phillips Jones


*For the London Medical and Physical Journal.*

### *Case of Strangulation of a Portion of the Intestines from Rupture of the Mesentery;* by Richard Phillips Jones, Esq.

A BOY, eight years of age, upon his return home in the evening from school, had some nuts given to him, which he cracked and ate: afterwards, he exercised himself with a skipping rope to such an excess we seldom hear of; the extent of which feat the little sufferer boasted of until he was put to bed. About twelve o'clock, he was suddenly attacked with most violent pains in the abdomen, resembling severe colic pains. Some cordial purgatives and other medicines were administered, and several times repeated, without producing any mitigation of the symptoms. The boy died in a very few hours from the commencement of the pains.

The body was examined three days after death. I observed no peculiarity in looking upon the corpse externally, save a considerable tumefaction of the abdomen, and which, upon compression, yielded with an unusual degree of elasticity. Having laid open the cavity of the abdomen, the following were the appearances:—One half of the small intestines occupying the right side were of an uniform deep purple hue, distended with flatus, and approaching in some parts to gangrene. The convolutions upon the left were, on the contrary, in the natural state, but were distended with flatus. The colon had entirely lost its character: so shrivelled up and contracted were the coats of this intestine, that I was even frustrated in the attempt to introduce the tip of my little finger within its cylinder; a considerable quantity of bloody-coloured serum was effused within the abdominal cavity. The stomach and duodenum were perfectly healthy; so was also the liver and all the solid viscera. The gall-bladder was very considerably distended. I was unable, for a time, what to make of the mesentery in this dissection. Being very desirous to have as simple a statement of the cause of death as possible, I very carefully unravelled the whole, and found precisely one half of the intestines to have passed through an opening at the root of the mesentery. The opening was so small as would, critically, allow nothing more to pass than intestine, drawing with it its portion of mesentery; assisted, probably, by the peristaltic motion of the intestines, as well as being urged by the violence of the exercise. Immediately in contact with the stricture, I found a hard substance wedged within the opening and within the intestine: so firmly was it fitted in this situation, that I could scarcely pass a probe between it and the sides of the stricture. I opened the gut to ascertain of what substance this ball was constituted: I found it to consist of the undigested nut-kernels the little boy had eaten; which, no doubt, had been so firmly lodged here during his severe exercise, and which produced so violent a strangulation, such extensive gangrene, and consequences so abruptly fatal to the little sufferer.

The hole in the mesentery appeared to have existed for some time; and it is very probable that the intestine may have been noosed by it, in a short time after its formation; but still the natural actions were carried on, and the fecal matter was allowed to pass, until the intestines, being excited by the undigestible nuts and the great exertion of the boy, became strangulated. The nuts were allowed to enter the noose, but were prevented from passing out again, and consequently created great irritation; a state very analogous to an intestine in a herniary sac becoming strangulated in consequence of the passage of some hard feculent matter.

22, *Suffolk-street*, *London*;
*Dec.* 28, 1818.

